# The Mediating Effect of Healthcare Utilization on Livelihood Empowerment against Poverty (LEAP) and Health Security of Older Adults in Ghana: A Case Study on the Ga-West Municipality in Accra, Ghana

**DOI:** 10.3390/healthcare10020370

**Published:** 2022-02-14

**Authors:** Edwina Naa Amerley Amarteifio, Baozhen Dai, Jonathan Aseye Nutakor, Ebenezer Larnyo

**Affiliations:** Department of Health Policy and Management, School of Management, Jiangsu University, 301 Xuefu Road, Zhenjiang 212013, China; amerley.amarteifio21@gmail.com (E.N.A.A.); jnutakor@gmail.com (J.A.N.); dr.ebenlarnyo@ujs.edu.cn (E.L.)

**Keywords:** old age, health utilization, health services, health security, LEAP

## Abstract

The concept of health security has aroused several interpretations because of theoretical technique indifferences or academic methodology. The focus has been on human security, old age health security (OAHS), whereas there remain issues of low healthcare utilization by older people from rural Ghana while there are social assistance programs. The study aimed at investigating the mediating effect of healthcare utilization on Livelihood Empowerment against Poverty (LEAP) and old age health security OAHS. With purposive sampling technique, participants were selected to participate in the study with standardized quantitative questionnaire to measure the variables involved in the study and a regression technique to analyze the data. The result of the mediation analysis showed a partial mediation between LEAP and Healthcare Utilization (HU) was found to have bridged the gap between the LEAP and OAHS. The LEAP policy also caused an increase in Health Utilization and, subsequently, an increase in old age health security (OAHS). The study is essential to help the National Health Insurance Authority NHIA in restructuring health care premiums to incite utilization of health facilities by the aged.

## 1. Introduction

The concept of health security has aroused several interpretations and conceptions because of indifferences in theoretical and academic approaches. The paradigm focus has been on human security until a shift in 2001. The paradigm shift in 2001 linked health security notion to a global approach for ameliorating the transmission of contagious illness throughout national borders [1]. This has called for a pragmatic approach to ensure that individuals, particularly the older population (above 60 years), are significantly cared for by instituting and implementing social policies that promote their health security.

The world has recently experienced about 3.2% estimated growth of old people above 60 years and will subsequently follow that trend in years to come [2]. Inarguably, 2015 saw a total world population of 900 million old people aged 60 years and over and it is assumed to exceed 2 billion by the year 2050 [3]. In Africa, it is estimated that by the year 2050, the number of older persons would rise to 161 million [2]. However, the aging population of Ghana is increasing exponentially, surpassing all other Sub-Saharan African countries [4]. Undoubtedly, the increase in the aging population means a huge burden on health care systems and facilities due to old age-associated diseases [5]. As a result, seeking frequent health care services would be the only panacea for these health challenges, hence the need for health security in the form of social interventions for those who cannot afford it.

Presently, social protection is principally about protecting the very poor and defenseless [6]. International organizations are driving social protection interventions with the International Labor Organization [7] initiating a Social Protection Floor (SPF), requiring member countries to “ensure an all-inclusive and universal program and policies for the extremely poor and vulnerable in societies”.

Old Age Health Security (OAHS) in Ghana aims to improve elderly health conditions, including Health Insurance Scheme and Livelihood Empowerment against Poverty (LEAP). The cardinal inspiration for adopting the LEAP was from the 2007 Ghana Statistical Service reportage on the number of abysmal homes in Ghana underutilizing healthcare (880,000, 18.2% of the population) [8]. In Ghana, LEAP is a pro-poor and social protection policy designed for the poor and venerable. This program has supported thousands of poor old adults in the communities. A study by Handa et al. [9] and Peprah et al. [6] reveals that the LEAP program offers monetary assistance to impoverished households, provides free enrollment into the NHIS for beneficiaries [9] which improves formal healthcare utilization [10].

The program since its inception in 2008 has focused greatly on giving cash transfers and training to these poor aged. The LEAP program provides cash transfers to poor households, orphans, persons with disabilities, and the older adults above 65 years Since its inception, the programs’ impact on local economic development, health and basic food needs has been tremendous [6]. It has encouraged the majority of the poor older adults to seek healthcare, and obtain health security. Debrah [8] asserts that LEAP beneficiaries who registered for National Health Insurance Scheme, especially pregnant women and the aged gained access to antenatal care and free health care. His findings show that, generally, LEAP fuels access to health care utilization.

The program has been described as a unique one that has supported livelihood, healthcare utilization and health security of the extremely poor and marginalized groups in Ghana. Currently, the LEAP program has reached over 35,000 households with an annual expenditure of approximately $11,000,000; bi-monthly expenses stretching from GHȻ 48.00 to GHȻ 90.00. LEAP has had a great impact on the lives of the beneficiaries and community developments [9].

Notwithstanding the global increase in literature on the health security of the areas above, there have been enormous bottlenecks to the operation of social intervention programs and the impact of social interventions on OAHS. In the same vein, there has recently been low utilization of healthcare services by older people in rural Ghana. It is against this backdrop that the present study examines the mechanisms through which the key social interventions such as the Livelihood Empowerment Against Poverty rolled out in Ghana contributes to Health Security of Older Adults through the mediation role of healthcare utilization.

## 2. Literature Review and Hypotheses Development

Alleviating poverty and promoting healthcare utilization has been a major issue of discussion by several international organizations. As a result, LEAP was instituted in Ghana. The cardinal inspiration for adopting the LEAP was from the 2007 Ghana Statistical Service reportage on the number of abysmal homes in Ghana underutilizing healthcare (880,000, 18.2% of the population) [8].

Previous studies have attempted to unravel the factors that stimulate access to healthcare utilization. Andersen et al. [11] stipulates several factors that incite citizenry to utilize healthcare services.

Other studies by Agyemang-Duah et al. [12] and Hwang et al. [13] concentrated primarily on LEAP and Old Age Health Security. To them, the LEAP program tends to boost older people’s health security in Ghana, but, Agyemang-Duah et al. [12] listed some hurdles to the LEAP in the quest to ensure older people’s health security. Contrary to Handa et al. [9], Agyemang-Duah et al. [14] reveal how the poor and elderly benefiting from LEAP are mandated to bear part of some medical expenses. Obviously, poor older people without financial strength will be unable to adequately use the healthcare services. To this end, they argue that removing financial barriers that inhibit access to professional healthcare under the LEAP is a turning point in the health of the older population because this positively influences formal healthcare utilization.

There is an increasing recognition worldwide of the necessity to appraise healthcare services’ usage and improve healthcare systems concerning the health needs of the aging populace [15]. Andersen Newman’s healthcare utilization model explored varied causalities that compel the use of health services by the aged. Basically, healthcare utilization means ‘obtaining healthcare from health service providers’ [16] Factors that incite a person’s tendency to use healthcare services include demographic and social characteristics such as gender, age, marital status, education level, living conditions, and children. The last factor from the Andersen healthcare utilization model is the need factor which reflects the illness level. Thus, one is compelled to utilize healthcare services due to health status, physical damage, changes in health status or having chronic diseases.

Zavras et al. [17] indicate that healthcare utilization depends on self-realized health status, age, income, gender, and religion. Precisely, we could argue that an increase in healthcare utilization means the availability of resources and patients’ level of satisfaction. This conforms to Liang et al.’s [18] findings that healthcare utilization improves older people’s psychological and general health.

The following hypotheses were developed:

**Hypotheses** **1** **(H1).** *There is a positive relationship between LEAP and Healthcare Utilization.*

**Hypotheses** **2** **(H2).** *There is a positive relationship between LEAP and OAHS.*

The aged require extensive healthcare as illness and disability increase due to aging [19]. The health security of older people in rural Ghana is a concern that prompted the establishment of the LEAP and the NHIS in 2004 by the Government of Ghana. The purpose was to guarantee equitable and widespread access for all Ghanaians to standard quality healthcare [20]. A study by the International Labor Organization [7] indicates that old people in low-and-middle-income countries are disadvantaged of a steady income because of lack of social pensions. This finding justifies the Government’s decision to exempt old people utilizing health care through the NHIS from paying annual premiums.

Notwithstanding the country’s profound demographic transition in adults’ quantity, it is critical to note that social interventions such as the NHIS and LEAP are vital to protect older people since their percentage is estimated to shift from 5% in 2015 to 10% in 2050 [2]. Blanchet et al. [21] noted that it is highly inequitable for an individual to make payments for healthcare since it acts as an obstacle to an individual acquiring timely and necessary treatment. More importantly, social interventions such as the LEAP and NHIS help increase healthcare utilization by old people. An increase in healthcare utilization means that the LEAP policy is yielding the desired results.

Based on the above, the study hypothesizes that:

**Hypotheses** **3** **(H3).** *There is a positive relationship between Healthcare Utilization and OAHS.*

**Hypotheses** **4** **(H4).** *Healthcare Utilization mediates the relationship between LEAP and OAHS.*

The World Health Organization [22] links health security to communicable disease control. However, WHO’s definition of health security raised some questions from authors such as The Lancet [23], who believed the organization had not entirely covered the bigger question in its definition, scope and implementation. Earlier, Ray-Bennett et al. [24] viewed health security as community participation, self-reliance and safeguarding of weak sects such as the aged, pregnant women and the poor, but Aldis et al. [1] argue that it’s detached from the regular public health tactics.

### Conceptual Framework

**Figure 1:**
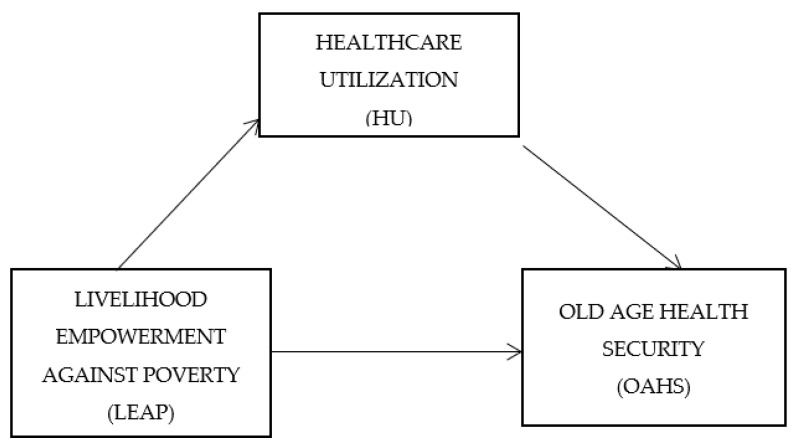
Conceptual model.

The conceptual framework in Figure 1 below was developed based on the literature review.

## 3. Materials and Methods

### 3.1. Questionnaire Design, Sampling and Data Collection

The study used the survey method, with structured questionnaire as the main instrument to collect data from old residents in the Ga West Municipal Assembly. The Ga-West Municipal Assembly is among the 29 Metropolitan, Municipal and District Assemblies (MMDA) in Greater Accra Region, with a population size of 219,788 [8]. The study selected the Amasaman Township from this Municipality purposively due to the high number of LEAP beneficiaries in the community. To comply with research ethics, we first sought permission from the District Chief Executive (DCE), Assembly man, Chief and elders of the area and finally the consent of respondents and the aim of the research communicated to them.

The Amasaman Township was purposively selected due to the high number of recorded LEAP beneficiaries in the community (1540). Based on this population size, a total sample size of 318 was estimated using a sample size estimation formula by Yamani (1967) as follows:

Sample size,

$$n=\frac{N}{1+N{\left(e\right)}^{2}}$$

where *N* is the total target population, *e* is the error margin. An error margin of 0.05 was proposed for the study hence the sample size was estimated as:

Sample size,

$$n=\frac{1540}{1+1540{\left(0.05\right)}^{2}}$$

= 318 participants.

Studying only older adults benefitting from the Livelihood Empowerment against Poverty Program, the study considered only those above 60 years who have lived in the study area for the past 10 years. However, since there was quite a number of older adults within the enclaves of the study area, the study first employed the purposive sampling technique using both inclusion and exclusion criterion to set the intended study respondents apart from others. Even though there is a tendency to commit a sampling bias using purposive sampling technique, the possible bias was minimized due to the inclusion and exclusion criteria used during the sampling.

From the sampled set, questionnaires were then randomly distributed community to community by the two research assistants following no particular order in order to represent the whole population. The 2 research assistants were not obliged to go back to households because a legible respondent was absent at the time of data collection. Once the target number was achieved, questionnaire distributions ceased.

The questionnaire had three sections: A, B, and C. Section “A” dealt with personal data of the respondents; section “B” consisted questions from related literature.; section “C” contained questions measuring the various variables (LEAP, Health Utilization and Old Age Health security). Section C was structured on a 5 point Likert scale, ranging from “Strongly Disagree” (SD), “Disagree” (D), “Neutral” (N), and “Agree” (A) to “Strongly Agree”. Respondents were instructed to respond according to their degree of agreement with the statements contained in the instrument(see Appendix A, Table A1)

Questionnaires were developed in simple plain English taking the ages of the respondents into consideration. They were administered face to face. Those who could not read or write were assisted by 2 research assistants who helped with translations. Those who could read but couldn’t write were still offered assistance to fill in the questionnaires.

A total of 1 month was used to distribute and collect data. Out of the 318 sampled respondents administered with questionnaires, only 307 answered questionnaires were returned; this represents 96.5% of the recovery rate of the sampling tool. Out of the 307 received, seven were incomplete so were regarded null. The response rate was good as the study covered majority of questionnaires distributed, representing a 94% response rate. Researchers have asserted that a high response rate, precisely above 60–70% is vital in ensuring accuracy in results [25,26].

### 3.2. Inclusion and Exclusion Criteria

The study sampled both elderly males and females in the age range of 61 years and above and had lived in the Municipality for the past 10 years. Individuals outside the age range and had not lived there for the expected years were excluded from the survey.

### 3.3. Measures

#### Result Variables

The outcome variable was old age health security (OAHS). This was initially measured on a 5-point Likert scale and then transformed into a continuous variable using the average responses. This variable measures the health security of the older adults. The key Predictor Variables were health utilization (HU) and the Livelihood Empowerment Program (LEAP). These Variables were also initially measured on a 5-point Likert scale and later transformed into a continuous variable using the average responses. The study used age, sex, marital status and occupational status as control variables in the regression analysis.

### 3.4. Statistical Analysis

The primary cross-sectional data collected were coded and analyzed using SPSS (version 23.0, IBM Corp., Armonk, NY, USA; 2015).

The study employed multiple statistical analyses approach which included descriptive statistical procedures, bivariate correlation, multivariate regression and mediation analysis using the Sobel test. Hypotheses were tested using the multivariate regression with OLS and the Sobel test of mediation effect. The study utilized the OLS approach to estimate the regression Equations (1) and (2). These regression models estimated both the direct and mediation effects of the Healthcare Utilization (HU) in the relationship between LEAP and Old Age health Security (OAHS) using the Sobel test. The regression models estimating the mediation effects were given as:

(1) $$M=\beta X+\varepsilon_{1}$$

(2) $$Y=\beta X+\beta M+\varepsilon_{3}$$

where Y denotes the main dependent variable, M represents the mediating variables and X also represents the independent variable. From equations, β is the coefficient of the explanatory variables while ε also denotes the error term. The Equation (1) estimates the direct impact of the independent variable (X) on the Mediating variable (M) while Equation (2) also estimates the direct impact of the explanatory variable (X) and mediator (M) on the dependent Variables (Y). Finally, the mediation effect was predicted using the Sobel Test. For mediation effect to exist, X must have significant direct impact of Y and M. Similarly, M must also have significant impact on Y to permit for the mediation analysis to be conducted.

The reliability of the data was checked using the Cronbach’s Alpha test where a cronbach alpha value of 0.7 and above implies strongly reliable variable construct. Likewise, the Bivriate Pearson correlation analysis was used to determine the strength and direction of relationships between the key interest variables; Livelihood Empowerment against Poverty (LEAP), Healthcare utilization (HU) and Old Age Health Utilization (OAHS). Finally, multi-collinearity test was performed using the Variance Inflation Factors (VIF) and tolerance where variables with VIF values less than 10 indicate the non-existence of multi-collinearity.

## 4. Results

### 4.1. Descriptive Statistics

The research continues the analysis by identifying the descriptive statistics of the data. The following table shows the descriptive characteristics of the variables in the data set and the demographic characteristics of respondents. The characteristics in the table display the general distribution of data variables. The total number of observations for the study was 300 with OAHS indicating its largest mean, showing the positive relationship LEAP has with the dependent variable as far as this study is concerned.

Of 300 participants, 101(33.6%) were between the ages of 61–65, the highest of the age groups. 111(36.9%) were males and 189 (62.9%). For occupation split by age groups, 45 (15.0%) were private workers, 99 (32.6%) were unemployed, 157 (52.2%) were pensioners enjoying pension benefits. Seventy-six (26.2%) were married, 72 (23.9) were widows and widowers, 90 (29.9%) divorced as shown in Table 1. The age group is expected due to the study under discussion, as the LEAP program mostly concentrates on the aged. In addition, the study focuses on OAHS, which makes the outcome on the age grouping so necessary for further analysis and discussion. The majority (44%) of respondents have first degrees as presented in Table 1.

### 4.2. Reliability Test

The Cronbach alpha obtained for the instrument was 0.984, indicating an excellent reliability based on the assertion of Blumberg et al. [27]. Results for the reliability test for the constructs are presented in Table 2.

### 4.3. Correlation Analysis

Pearson correlation analysis was used to determine the strength and direction of relationships. Table 3 shows a positive correlation between the dependent variable, mediating and independent variable. Displayed variables are Livelihood Empowerment against Poverty (LEAP), Healthcare Utilization (HU) and Old Age Health Security (OAHS). Table 3 shows a positive correlation between the dependent variable, mediating and independent variable. Results from Table 3 reveal a strong positive and statistically significant relationship between LEAP and OAHS (*r* = 0.956, *p* = 0.000). This implies that, as LEAP experiences a considerable improvement possibly as more cash has been transferred to beneficiaries, there is a high tendency that their health security also improves. Similarly, results also indicate that LEAP has positive and statistically significant relationship with the HU (*r* = 0.576, *p* = 0.000). The implication is that the two variables increase together; however, there is moderate association between the variables.

Moreover, HU has a statistically significant and positive effect on OAHS, (*r* = 0.928, *p* = 0.000). This also suggests that Healthcare Utilization tends to have a strong association with the health security of old age. An improvement in access to healthcare services is highly linked to improved access to health security during old age.

### 4.4. Regression Analysis

The multivariate regression analysis was utilized to examine the extent to which the Livelihood Empowerment Program (LEAP) and healthcare utilization influence older adults health security. The results from regression Model 1 focused on testing Hypothesis 1 (H1) which sought to examine the impact of LEAP on Healthcare Utilization among the older adults. The results from Table 4 indicate an R-square value of 0.783 (R^2^ = 0.783) which implies that, about 78.3% of the variations in the dependent variables (Healthcare Utilization) is explained by the independent variables such as LEAP, and the control variables such as marital status and occupation of the respondents. Similarly, results from Table 4 shows that, the F-statistics (F = 104.153, *p* < 0.001) was statistically significant at 1% level. This implies that, the independent variables have joint statistically significant impact on the dependent variable (Healthcare Utilization).

Findings from regression model 1 in Table 4 reveal that, LEAP has a statistically significant and positive impact on Healthcare Utilization (β = 0.759, *p* < 0.001), meaning an improvement in the Livelihood empowerment program increases the healthcare utilization among older adults. These results support Hypothesis 1 (H1) which states that, there is a positive relationship between LEAP and Healthcare Utilization.

The results from Table 4 also indicate that some demographic factors such as marital status and occupation have statistically significant positive impact on healthcare utilization among respondents. The results show that Divorced respondents (β = 0.141, *p* < 0.10) are more likely to utilize healthcare services compared to the Unmarried. In terms of occupation of respondents, the results show that being a private worker (β = 0.215, *p* < 0.05) has a significant positive impact on healthcare utilization compared to the unemployed (see Table 4).

Regression Model 2 in Table 5 sought to test the research Hypotheses 2 and 3. The analysis emphasizes on examining the impact of LEAP and Healthcare Utilization on Older Adults Health Security (OAHS). Results obtained present an R-square value of 0.957 (R^2^ = 0.957) which implies that, about 95.7% of the variations in the dependent variables (OAHS) are explained by the independent variables such as LEAP, Healthcare Utilization and some demographic factors such as Age, sex, marital status and occupation. The results from Table 5 also shows that, the F-statistics (F = 580, *p* < 0.001) was statistically significant at 1% level. This means that the independent variables jointly influence the dependent variable (OAHS) which was statistically significant at 1% level.

The regression model 2 in Table 5 indicates that, LEAP (β = 0.650, *p* < 0.001) has a statistically significant and positive direct impact on OAHS. This supports the research Hypothesis 2 (H2) which states that there is a positive relationship between LEAP and OAHS. This result implies that, an improvement in the Livelihood empowerment program tend to promote the older adults’ health security.

Moreover, the results from Table 5 depicts that, Healthcare Utilization (β = 0.481, *p* < 0.001) has statistically significant positive impact on the older adults’ health security. This finding also supports research Hypothesis 3 (H3) which states that there is a positive relationship between Healthcare Utilization and OAHS.

Results from Table 5 further show that, some demographic factors such as age of respondents has positive impact on their health security. It shows that older adults aged 70–75 years (β = 0.078, *p* < 0.05) are more likely to positively influence their health security compared to those who are 61–65 years. The results also show that respondents who were Divorced (β = 0.138, *p* < 0.01) and those who were married (β = 0.123, *p* < 0.01) are more likely to have health security at their old age compared to the respondents who were Unmarried. In terms of occupation of respondents, being a private worker (β = −0.108 **, *p* < 0.05) has a significant negative impact on healthcare security compared to the unemployed (see Table 4). However, being a pensioner (β = 0.086, *p* < 0.05) has a positive impact on the health security compared to the unemployed (see Table 5).

Table 6 displays the collinearity statistics with variables showing multi-collinearity non-existence as all the Variance Inflation Factors (VIF) is less than five.

### 4.5. Mediation Analysis

To check the mediating effect of healthcare utilization, the study adopted the Sobel test analysis. This is to test the mediating criteria and to assess whether indirect effects were significant or not. Table 7 presents the outcome.

Results from the Sobel test revealed that the complete pathway from LEAP (independent variable) to HU (mediator) to OAHS (dependent variable) was significant (z = 12.94, *p* = 0.000). Hence, HU partially mediates the effects of both the independent and dependent variables. The results also indicate that the independent variable contributes directly to explaining the variation on Old Age Health Security. The test confirms the effect of HU as a mediator between the independent and dependent variables thus, supporting H4.

## 5. Discussion

The foremost goal of the paper was to examine the mediating role of HU on LEAP and OAHS. Results from the sobel test from Table 7 confirmed the effect of HU as a partial mediator between the independent and dependent variables; thus, this objective was achieved. Although there is a countless number of literature on old age health security, there remains a gap in the link between social intervention programs and old age health security. This necessitated the need to look into this area of the study. Practically, cash transfers have been the main modus operandi for most developing countries that implement social protection policies. It is the same with the implementation of Ghana’s LEAP policy.

Studies have shown that healthcare utilization among old age increases as their ages also keep increasing. It is hence, expected that with the LEAP supporting old age health expenses, there should be a majority of old age healthcare utilization. The results from the study show a positive strength between LEAP and healthcare utilization. This finding confirms the discoveries of Appiah et al. [28] and Appiah et al. [29]. The scholars embarked on the predictors of healthcare utilization among poor older people under the LEAP program in the Atwima Nwabiagya District of Ghana.

However, their work further reveals that barriers associated with low utilization of healthcare services bore down to financial and proximity contemplations. The prevailing barriers were also highlighted in the works of Bhan et al. [30], mentioning financial constraints, inadequate health professionals and facilities. However, low utilization of healthcare utilization is a menace to old age health security.

The importance of healthcare utilization is highlighted in the work of Atchessi et al. [31], which revealed that about 53% of older people who utilize health facilities are boosted by the presence of health insurance. In affirmation, Awoke et al. [32], in their cross-sectional survey in Ghana, found an increase of 17.8% and 51.7% of old people utilizing private and public healthcare facilities, respectively, due to LEAP program. From the above, Agyemang-Duah et al. [14] assertion that LEAP offers support for the aged to utilize healthcare services cannot be underestimated.

Moreover, the prevalence of healthcare utilization is high in Ghana and rural sub-Sahara African settings [33]. This increase in developed countries is undoubted as numerous health insurances are prevalent in those regions.

The study also discovered a substantial association between LEAP and old age health security. It revealed the participants’ belief in LEAP being the bedrock for old age health security. This observation convinced the researcher to accept the earlier assumption. Specifically, the elderly who suffer from chronic diseases are likely to utilize healthcare services more often. Their postulation is in line with Hajek et al. [33], which revealed a substantial link between chronic disease and healthcare utilization. In developing countries such as Ghana, the LEAP has become a cornerstone for poverty reduction and old age health security. The researches of Peprah et al. [6] and Handa et al. [9] reveal that the LEAP program provides financial support to extremely poor family units including older people seeking healthcare. Dai [34] posited that in rural China, social health insurance scheme is one vital constituent of rural folks who are old and need health security. To explicate, Baozhen mentioned the importance of the role insurance plays in ensuring that the elderly in rural areas can afford and access to basic healthcare services. It is significant to note that the old age health security in Ghana is one of the targets to promote good health among the age. The NHIS and the LEAP program have not failed to achieve this.

To promote the health of the old people, there should be a well-structured healthcare system that will aid in utilizing healthcare services. In other words, an efficient and reliable system is vital to the promotion of the health of old people. It is, however, not surprising as the observations of this research reveal an affirmative relation between healthcare utilization and old age health security.

Several studies have raised the need to educate old people on the need to utilize healthcare services to promote their health. This agrees with other empirical studies on the significant association between education and OAHS [5,35]. However, Mcnamara et al. [36], disagreed with the assertion that the more the older people are educated on their health status, the more they use healthcare facilities. They opined that elderly people’s level of use of healthcare facilities is not merely dependent on education.

Furthermore, results from the study prove that health utilization partially mediates the relationship between the LEAP and old age health security. The finding is relatively similar to that of Gyasi [37]. Their study asserts that higher utilization is a springboard to the health security of old people and this aid in enrolling a higher number of old people onto the LEAP program. In other words, higher utilization means higher enrollment in LEAP as well as a promotion of health security. The study, however, recognizes the impact the LEAP directly has on old age health security.

Consequently, the mediating effect of healthcare utilization is seen to have a significant association with LEAP and old age health security. This implies that in order to promote old age health, the LEAP programs need to be vibrant and promote healthcare utilization. A reduction in the smooth operation of the LEAP may directly or indirectly hinder the health status of old people.

In spite of the relevant contributions of this study, some limitations are associated with this study. Noting these limitations may help guide future studies to extend the knowledge in the study area and achieve more improvement in their results. Firstly, the unique characteristics of urban communities in a developing country used as the study area limit the broader generalization of the findings. The extent of availability of quality healthcare facilities, health security and the livelihoods in the urban communities are different from that of communities in the rural areas and other jurisdictions. It is therefore important for future studies to consider other peri-urban communities in the rural settings. Again, results implied a causal relationship between LEAP, OAHS and HU. However, the use of the cross-sectional data and the type of research design employed limits the ability of the study to draw a solid conclusion on the causal relationship between the variables. It is possible there may be backward causation between HU and AOHS. Nonetheless, our data and research design does not permit such test. Hence future use of longitudinal data would be more appropriate to test and establish whether there is a reverse strong causality between these variables. Further, our sample size was particularly limited coupled with self-reported data.

Future study may expand the sample size and incorporate a more rigorous statistical computation approach to address the robustness of the mediation analysis. It could also consider adding more moderating variables such as socio-demographic features of the participants and could also focus of comparative study between the urban and the rural communities in other countries to expand the scope of the study.

## 6. Conclusions 

The study explores a model that unravels the mediation of healthcare utilization on the relationship between LEAP and old age health security. It was found out that the LEAP program has made considerable strides in healthcare promotion among the more aging population.

The results additionally indicated a positive relationship between Livelihood Empowerment against Poverty (LEAP), Healthcare Utilization (HU), and Old Age Health security (OAHS). Healthcare Utilization (HU) was found to have bridged the gap between the LEAP and OAHS. The LEAP policy caused an increase in Health Utilization and, subsequently, an increase in old age health security (OAHS). The result from the mediating analysis showed the complete pathway from LEAP to HU to OAHS was significant (z = 12.94, *p* = 0.000), indicating that HU partially mediates the effects of the independent variable and the dependent variable.

Our results demonstrate that, the livelihood and empowerment against poverty program which provides monthly cash transfers to the older adults plays a significant role in supporting the older adults to access the healthcare facilities in the communities. This program has both direct and indirect significant positive influence on the older adults’ health Security. The older adults are the groups that have been identified to have high rate of healthcare utilization due to their health status needs, susceptibility to frequent illness, weak immune systems, and high risk to falling sick. Through the LEAP program, the results show that, their health security has also been improved.

The findings from this study therefore offers a strong implication for extended empirical and theoretical understanding of social support systems, healthcare utilization, wellness, health among the older adults from a developing country’s perspective. The findings further provide support and guide to inform relevant policy on health and ageing, healthcare utilization and health security of the population. These findings from the study also inform a new direction of research in the areas of ageing and healthcare utilization and individual health outcome studies in the developing countries.

The study recommends that there is a need for more efforts to maintain the LEAP program. Future studies may focus on the impact of Ghana’s National Health Insurance Scheme on old age health security. It is finally recommended that local governments mobilize resources from their localities and distribute the LEAP funds to the beneficiaries instead of depending on central governments to avoid a delay in disbursement.

## Figures and Tables

**Table 1:** Descriptive Statistics of variables.

Variable	Mean	Std. Deviation	N
Older Adults Health Security(OAHS)	2.494	1.150	300
LEAP	2.487	1.071	300
Healthcare Utilization	2.416	0.987	300
Demographic characteristics		Frequency	Percentage (%)
Age	61–65	101	33.7
	66–70	92	30.7
	71–75	71	23.7
	76 and above	36	12.0
Gender	Male	111	37.0
	Female	189	63.0
Marital Status	Married	79	26.3
	Widowed	72	24.0
	Unmarried	59	19.7
	Divorced	90	30.0
Occupation	Private Sector	45	15.0
	Unemployed	98	32.7
	Pensioner	157	52.3
Level of Education	Secondary	59	19.7
	Vocational	98	32.7
	Tertiary	132	44
	Masters	11	3.6

**Table 2:** Reliability Statistics of variables.

Description	Cronbach’s Alpha	Number of Items
LEAP	0.969	7
Healthcare Utilization	0.935	6
Old Age Health Security	0.988	6
Overall Questionnaire Reliability	0.984	17

**Table 3:** Results of Correlation analysis.

		OAHS	LEAP	HU
Pearson Correlation	OAHS	1.000	0.956	0.928
	LEAP	0.956	1.000	0.576
	HU	0.928	0.576	1.000
N	OAHS	300	300	300
	LEAP	300	300	300
	HU	300	300	300

**Table 4:** Regression Model 1 (Dependent Variable: Healthcare Utilization).

Variable	B	Std. Error	t	*p*-Value
LEAP	0.759 ***	0.029	26.467	0.000
Age				
61–65	Reference			
66–70	0.078	0.070	1.113	0.266
71–75	0.028	0.076	0.365	0.715
76 and above	0.014	0.096	0.144	0.885
Sex				
Male	Reference			
Female	−0.026	0.063	−0.412	0.681
Marital status				
Unmarried	Reference			
Married	−0.065	0.087	−0.746	0.456
Widowed	0.064	0.087	0.733	0.464
Divorced	0.141 *	0.081	1.734	0.084
Occupation				
Unemployed	Reference			
private worker	0.215 **	0.094	2.302	0.022
Pensioner	−0.086	0.064	−1.343	0.180
(Constant)	0.485 ***	0.110	4.401	0.000
R-square	0.783			
Ad. R-Square	0.775			
*F* (104.153, *df* =11)	*p* < 0.001			

Note: *** *p* < 0.001, ** *p* < 0.05 and * *p* < 0.10.

**Table 5:** Regression Model 2: (Dependent Variable: Older Adults Health Security).

Variable	B	Std. Error	t	*p*-Value
LEAP	0.650 ***	0.028	23.553	0.000
Healthcare Utilization	0.481 ***	0.031	15.706	0.000
Age				
61–65	Reference			
66–70	0.008	0.036	0.232	0.816
71–75	0.078 **	0.039	1.982	0.048
76 and above	0.068	0.050	1.358	0.176
Sex				
Male	Reference			
Female	−0.052	0.033	−1.600	0.111
Marital status				
Unmarried	Reference			
Married	0.123 ***	0.045	2.737	0.007
Widowed	−0.040	0.045	−0.880	0.380
Divorced	0.138 ***	0.043	3.233	0.001
Occupation				
Unemployed	Reference			
private worker	−0.108 **	0.049	−2.205	0.028
Pensioner	0.086 **	0.033	2.572	0.011
(Constant)	−0.372 ***	0.059	−6.288	0.000
R-Square	0.957			
Ad. R-Square	0.955			
*F* (580, *df* = 11)	*p* < 0.001			

Note: *** *p* < 0.001, ** *p* < 0.05.

**Table 6:** Multi-collinearity Test.

Model		Collinearity Statistics	
		Tolerance	VIF
1	LEAP	0.233	4.283
	HU	0.233	4.283

Dependent Variable: OAHS.

**Table 7:** Sobel Test of Mediation.

Independent Variable	Test Statistics(z)	Std. Error	*p*-Value
LEAP	12.94	0.03	0.000

## Data Availability

The data presented in this study are available on request from the first and corresponding author.

## Appendix A

**Table A1:** Questionnaire items and responses related to LEAP, HU and OAHS.

Items	Percentages of Responses				
	Strongly Disagree%	Disagree%	Neutral%	Agree%	Strongly Agree%
Statements measuring LEAP					
I am a LEAP beneficiary				45	55
I am covered by LEAP because I cannot afford health services				31.3	68.7
Leap facilitates my access to complementary health services				5.7	94.3
LEAP encourages healthcare utilization	4.3	7	17	38.7	33
I utilize healthcare services under LEAP more than I did 5 years ago without LEAP			3.3	65.3	31.3
Leap Program also encourages me to seek healthcare for my own health security				70.7	29.3
Statements on Old age Health Security					
I am very confident of my Health status	7.3	17.3	15.7	32.7	27
I receive quality health service from LEAP just like any other healthcare providers		10.7	15.7	58.6	15
I am responsible for my health security		4.3	2.7	52	41
I report the slightest health issues I experience to secure my health				70.7	29.3
Social intervention programs are necessary for my health security		6	8	49.3	36.7
Social intervention programs do not assure my health security		59	20.7	11.3	9
Statements on health utilization					
I often utilize health services	0.4	7	21	30.6	41
healthcare services are available but not within my reach	3	4	3	29	61
I utilize healthcare services because of chronic diseases that come with my age		5	9	2.3	83.7
I utilize health services to secure my health			2	42	56
I often go for checkups whether sick or not	36.8	14	7	26.7	15.5
I utilize healthcare services under LEAP more than I did 5 years ago without LEAP			6.4	28.3	65.3
I utilize health services because of my health beliefs		8.3	37	22	32.7
Responses on the presence of healthcare utilization on LEAP and OAHS					
I can secure my health if I make use of Health services made available		4.4	20.3	17.6	57.7
I can’t benefit from leap if I am not able and willing to utilize health services	1.5		8.3	23	67.2
Utilizing health services require financial support covered by leap and health insurance			12..1	53.6	34.3
An operational Leap is promoted by utilizing healthcare which ensures health security		0.7	34.3	16	49
Utilizing healthcare services is the core of securing one’s health			4.2	54.5	41.3

## References

[1] Aldis W. (2008). Health security as a public health concept: A critical analysis. Health Policy Plan..

[2] United Nations (2015). World population prospects: The 2015 revision. United Nations Econ. Soc. Aff..

[3] He W., Goodkind D., Kowal P. (2016). An Aging World: 2015 (International Population Reports, P95/16-1).

[4] Mba C.J. (2010). Population ageing in ghana: Research gaps and the way forward. J. Aging Res..

[5] Bauman A., Merom D., Bull F.C., Buchner D.M., Fiatarone Singh M.A. (2016). Updating the evidence for physical activity: Summative reviews of the epidemiological evidence, prevalence, and interventions to promote “active aging”. Gerontologist.

[6] Peprah P., Kyiyaga E.M., Afful H., Abalo E.M., Agyemang-Duah W. (2017). Does the Ghanaian livelihood empowerment against poverty programme lead to an increase in household productive livelihood assets?. Anal. Ashanti Scenar..

[7] International Labour Organization (2012). Social Protection Floor for a Fair and Inclusive Globalization.

[8] Debrah E.J.A.T. (2013). Alleviating poverty in Ghana: The case of livelihood empowerment against poverty (LEAP). Afr. Today.

[9] Handa S., Park M., Darko R.O., Osei-Akoto I., Davis B., Daidone S. (2013). Livelihood Empowerment Against Poverty Program Impact Evaluation.

[10] Sackey P.-K., Remoaldo P.J.C.S.S. (2019). Ghana’s Livelihood Empowerment Against Poverty (LEAP) programme is leaking: Irregularities watering down the impact of the flagship LEAP programme. Cogent Soc. Sci..

[11] Andersen R.M. (1995). Revisiting the behavioral model and access to medical care: Does it matter?. J. Health Soc. Behav..

[12] Agyemang-Duah W., Peprah C., Peprah P. (2019). Barriers to formal healthcare utilisation among poor older people under the livelihood empowerment against poverty programme in the Atwima Nwabiagya District of Ghana. J. BMC Public Health.

[13] Hwang K., Kulkareni S., Hu Y. Cloud security with virtualized defense and reputation-based trust mangement. Proceedings of the 2009 Eighth IEEE International Conference on Dependable, Autonomic and Secure Computing.

[14] Agyemang-Duah W., Owusu-Ansah J.K., Peprah C. (2019). Factors influencing healthcare use among poor older females under the Livelihood Empowerment Against Poverty programme in Atwima Nwabiagya District, Ghana. J. BMC Res. Notes.

[15] Nie J.X., Wang L., Tracy C.S., Moineddin R., Upshur R.E. (2008). Health care service utilization among the elderly: Findings from the Study to Understand the Chronic Condition Experience of the Elderly and the Disabled (SUCCEED project). J. Eval. Clin. Pr..

[16] Jiang M., Yang G., Fang L., Wan J., Yang Y., Wang Y. (2018). Factors associated with healthcare utilization among community-dwelling elderly in Shanghai, China. PLoS ONE.

[17] Zavras D., Geitona M., Kyriopoulos J. (2014). Primary health services utilization in Greece: Studying the past for planning the future. Soc. Cohes. Dev..

[18] Liang Y., Lu P. (2014). Medical insurance policy organized by Chinese government and the health inequity of the elderly: Longitudinal comparison based on effect of New Cooperative Medical Scheme on health of rural elderly in 22 provinces and cities. Int. J. Equity Health.

[19] Van der Wielen N., Channon A.A., Falkingham J. (2018). Universal health coverage in the context of population ageing: What determines health insurance enrolment in rural Ghana?. BMC Public Health.

[20] Ying F., Dartey B., Gyabeng E. (2019). Ascertaining the Influence of Politics on the Outcomes of the Implementation of the NHIS: Study of the Ga West District Mutual Health Insurance Scheme. Public Policy Adm. Res..

[21] Blanchet N.J., Fink G., Osei-Akoto I. (2012). The effect of Ghana’s National Health Insurance Scheme on health care utilisation. Ghana Med. J..

[22] WHO (2007). Everybody’s Business—Strengthening Health Systems to Improve Health Outcomes: WHO’s Framework for Action.

[23] The Lancet (2007). WHO Fails to Address Health Security.

[24] Ray-Bennett N.S., Collins A., Bhuiya A., Edgeworth R., Nahar P., Alamgir F. (2010). Exploring the meaning of health security for disaster resilience through people’s perspectives in Bangladesh. Health Place.

[25] Babbie E.J.B. (2013). The Practice of Social Research (13th Student Ed.).

[26] Creswell J.W., Poth C.N. (2016). Qualitative Inquiry and Research Design: Choosing among Five Approaches.

[27] Blumberg C., Cooper D., Schindler S.J.B.I. (2008). Business Research Methods.

[28] Appiah J.O., Agyemang-Duah W., Fordjour A.A., Adei D. (2020). Predicting financial barriers to formal healthcare utilisation among poor older people under the Livelihood Empowerment Against Poverty Programme in Ghana. GeoJournal.

[29] Appiah J.O., Agyemang-Duah W., Peprah C., Adei D., Peprah P., Fordjour A.A. (2020). Transportation barriers to formal healthcare utilisation and associated factors among poor older people under a social protection programme in Ghana. J. Transp. Health.

[30] Bhan N., Madhira P., Muralidharan A., Kulkarni B., Murthy G.V.S., Basu S., Kinra S. (2017). Health needs, access to healthcare, and perceptions of ageing in an urbanizing community in India: A qualitative study. BMC Geriatr..

[31] Atchessi N., Ridde V., Abimbola S., Zunzunegui M.-V. (2018). Factors associated with the healthcare-seeking behaviour of older people in Nigeria. Arch. Gerontol. Geriatr..

[32] Awoke M.A., Negin J., Moller J., Farell P., Yawson A.E., Biritwum R.B., Kowal P. (2017). Predictors of public and private healthcare utilization and associated health system responsiveness among older adults in Ghana. Glob. Health Action.

[33] Hajek A., Bock J.-O., König H.-H. (2017). The role of personality in health care use: Results of a population-based longitudinal study in Germany. PLoS ONE.

[34] Dai B. (2015). The old age health security in rural China: Where to go?. Int. J. Equity Health.

[35] Tamayo-Fonseca N., Nolasco A., Quesada J.A., Pereyra-Zamora P., Melchor I., Moncho J., Calabuig J., Barona C. (2015). Self-rated health and hospital services use in the Spanish National Health System: A longitudinal study. BMC Health Serv. Res..

[36] McNamara C., Labonté R. (2017). Trade, labour markets and health: A prospective policy analysis of the Trans-Pacific Partnership. Int. J. Health Serv..

[37] Gyasi R.M. (2018). Ageing, Health and Health-Seeking Behaviour in Ghana.

